# Comparison of Psychosocial Variables Associated With Loneliness in Centenarian vs Elderly Populations in New Zealand

**DOI:** 10.1001/jamanetworkopen.2018.3880

**Published:** 2018-10-26

**Authors:** Sharon Leitch, Paul Glue, Andrew R. Gray, Philippa Greco, Yoram Barak

**Affiliations:** 1 Department of General Practice and Rural Health, Dunedin School of Medicine, University of Otago, Dunedin, New Zealand; 2 Department of Psychological Medicine, Dunedin School of Medicine, University of Otago, Dunedin, New Zealand; 3 Biostatistics Unit, Dunedin School of Medicine, University of Otago, Dunedin, New Zealand; 4 Southern District Health Board, Dunedin, New Zealand

## Abstract

**Importance:**

Loneliness is associated with reduced health-related quality of life and increased morbidity and mortality and typically worsens with aging.

**Objectives:**

To evaluate associations between demographic and psychosocial variables and loneliness, examine any age-specific associations, and compare centenarians (aged ≥100 years) with elderly people (aged 65-99 years).

**Design, Setting, and Participants:**

In this retrospective, observational, cross-sectional study, previously collected data from all New Zealanders 65 years and older who completed their first international Resident Assessment Instrument–Home Care (interRAI-HC) assessment during the study period (January 1, 2013, to November 27, 2017) were reviewed. Participants were people living independently in the community who were requesting or referred for assessment with a potential need for support services.

**Main Outcomes and Measures:**

The interRAI-HC is a 236-item, electronically recorded assessment that encompasses a comprehensive range of aspects of an older person’s life, including physical, psychological, and cognitive domains. Eight main items from the interRAI-HC data set were analyzed to describe the population and evaluate the core psychosocial components of aging, namely, age, sex, race/ethnicity, marital status, living arrangements, family support, depression, and loneliness. Loneliness was evaluated by the participants’ response to the assessment statement, “Says or indicates that he/she feels lonely.”

**Results:**

A total of 73 286 New Zealanders (mean age, 81.4 years; age range, 65-109 years; 41 641 [56.9%] female) participated in the study. The assessments of 191 centenarians (mean [SD] age, 100.9 [1.2] years) and 73 095 elderly people (mean [SD] age, 81.4 [7.6] years) were analyzed. Centenarians vs elderly people were more likely to be female (136 [71.2%] vs 41 488 [56.8%]; *P* < .001), and the populations differed by marital status (widowed: 170 [89.0%] vs 31 554 [43.2%]; overall *P* < .001) and depression status (70.2% vs 59.5% free of depression; overall *P* = .008). Centenarians were less likely to be lonely compared with elderly people, with a 22% lower risk of loneliness for a typical centenarian (aged 100.9 years) compared with a typical elderly person (aged 81.4 years) in unadjusted analyses (relative risk, 0.78; 95% CI, 0.67-0.92; *P* = .002). In the fully adjusted model, there was a 32% reduction in loneliness for a centenarian compared with an elderly person (relative risk, 0.68; 95% CI, 0.58-0.79; *P* < .001). Living with others, having family support, and lacking depression were associated with lower risk of loneliness.

**Conclusions and Relevance:**

Centenarians are a unique group to study as a model of successful aging. The sample of centenarians in this study appeared to be less lonely than other groups studied internationally. The study identified multiple psychosocial variables that were associated with the risk of loneliness, including living arrangements, family support, and depression. Knowing these variables may help our society address risk factors for loneliness in older people.

## Key Points

**Questions:**

Are centenarians less lonely than elderly people, and if so, are there any demographic and psychosocial differences that may account for this?

**Findings:**

This cross-sectional study of 73 286 community-dwelling New Zealanders 65 years and older found that centenarians were more likely to be female, widowed, living alone or with relatives, receiving family support, and not depressed compared with those aged 65 to 99 years. Loneliness was significantly less common with older age, and living arrangements, race/ethnicity, marital status, family support, and depression were significantly associated with loneliness.

**Meaning:**

Knowing the variables associated with loneliness may help our society address risk factors to reduce loneliness in older people.

## Teaser

This cross-sectional study of community-dwelling New Zealanders 65 years and older examines the association between loneliness and psychosocial variables in centenarians compared with elderly people.

## Introduction

Loneliness is associated with reduced quality of life and increased morbidity and mortality.^1,2^ Centenarians are a unique group to study as a model of successful aging; examination of their psychosocial demographics may help identify factors that reduce loneliness and its sequelae.

The number of centenarians has increased significantly since the 1980s in New Zealand. On the basis of 2011 data, an estimated 400 to 500 New Zealand centenarians are likely currently alive, with less than 10% of centenarians older than 105 years.^3^ Death rates among females are lower than those among males at all ages, resulting in a population ratio of approximately 1 man to 6 women in the centenarian age group.^3^

A study from New Zealand^4^ found that loneliness was reported by 21% of older New Zealanders; however, that study did not focus on centenarians. Several small international studies have found high levels of loneliness among centenarians: 44% of 400 Greek centenarians were lonely,^5^ 55% of 55 Portuguese centenarians were lonely,^6,7^ 31% of 78 centenarians in Georgia were lonely,^8^ and 44% of 77 Swedish centenarians were lonely.^9^

New Zealand is the first country in the world to implement the international Residential Assessment Instrument–Home Care (interRAI-HC) for all people who are being considered for access to publicly funded community services or entry into aged residential care, providing a rich data resource for researchers. The present study examined the association between loneliness and psychosocial variables from interRAI-HC assessments undertaken in a large sample of community-dwelling older people, including centenarians.

## Methods

### Participants

Participants in this study were New Zealanders 65 years and older who completed their first interRAI-HC assessment during the study period (January 1, 2013, to November 27, 2017). The interRAI assessments are undertaken in New Zealand for people 65 years or older with an age-related disability that is affecting their function, likely to continue for a minimum of 6 months, and creates a potential need for support services. If participants underwent multiple assessments during the study period, only data from the initial assessment were reviewed. Participant data were completely anonymized; no personal identifying data, such as National Health Index number or date of birth, were reviewed. Age was recorded at the time of assessment. Season was determined by assessment date (summer was defined as December through February and so on). Race/ethnicity was self-identified by participants or their authorized representatives, which is the standard method for reporting race/ethnicity in New Zealand.^10^ Collection of these data can help identify any underlying disparities attributable to race/ethnicity and may facilitate targeted strategies to improve health outcomes and reduce inequalities.^11^ This study followed the Strengthening the Reporting of Observational Studies in Epidemiology (STROBE) reporting guideline for cross-sectional studies.^12^ The Ngāi Tahu Research Consultation Committee was consulted about this study with regard to the consequences on Māori (the indigenous people of New Zealand). Participants or their authorized representatives were asked whether they consented for their anonymized information to be used for research purposes at the time of their interRAI-HC assessment; only the interRAI-HC data from informed consenting participants (96%) were reviewed. Ethical approval was obtained from the University of Otago Ethics Committee and the Department of Psychological Medicine Ethics Committee.

### Measurements

The interRAI is an evidence-based, 236-item, electronically recorded assessment tool.^13^ It encompasses a wide range of aspects of an older person’s life, including physical, psychological, and cognitive domains, to determine which services might best meet that person’s need. Eight main items from the interRAI-HC data set were analyzed in this initial exploration, including demographic items to describe the population (age, sex, and race/ethnicity) and items that reflected the core psychosocial components of aging (marital status, living arrangements, family support, depression, and loneliness).

Loneliness was evaluated by the participants’ response to the assessment statement, “Says or indicates that he/she feels lonely.” Self-reported loneliness has been reported to be longitudinally stable among older people.^14^ This simple interRAI-HC measure of loneliness is related to a variety of health outcomes, such as impairment of vision and hearing, cognition, and general functional difficulties.^15^

### Statistical Analysis

Descriptive statistics are presented for all variables, including demographics and health variables, by centenarian status and percentage of lonely individuals. The χ^2^ test was used to compare the distribution of categorical variables between centenarians and noncentenarians. Poisson regression with robust SEs^16^ was used to model associations with the binary outcome of loneliness and potential indicators (age being the variable of primary interest), estimating relative risks (RRs) for each (because loneliness is not a rare outcome and therefore odds ratios from logistic regression models would overestimate the RRs to a meaningful degree).

Initially, age as a categorical variable was investigated (midpoints from 65 to 105 years in 5-year increments). This approach demonstrates the shape of any association between loneliness and age without assuming its form but relies on the choice of cut points, is known to be statistically inefficient, and does not acknowledge the likely smooth transitions over ages in terms of the risk of loneliness^17^; therefore, it was used only to ensure that models using polynomials that involved age were not overfitting.

Next, univariable models were investigated, showing unadjusted associations between loneliness and each variable (age as a continuous variable, sex, race/ethnicity, season, marital status, family support, living arrangements, and depression). For the model with age, to model potential nonlinearities, separate regression models based on age; age and age squared; age, age squared, and age cubed; and age, age squared, age cubed, and age to the fourth power were investigated with the model with the lowest Bayesian information criterion selected. Then, a partially adjusted model with age (again as a polynomial), sex, race/ethnicity, and season was examined (these are all considered to be effectively nonmodifiable variables). Finally, a fully adjusted model that also included marital status, family living arrangements, family support, and depression was examined because these are all considered to be potentially modifiable factors. To assist interpretation of the models, linear contrasts were used to compare the RR of loneliness between individuals aged 65 to 99 years (mean age, 81.4 years) and those 100 years and older (mean age, 100.9 years).

To explore the potential modification of the association of age with each of the other variables (eg, whether any association with age varied by sex), interactions were added to the fully adjusted model and retained if statistically significant. Because of the small number of centenarians, some variables were collapsed for this purpose. The same approach was used for sex with each of marital status, living arrangements, and family support.

Missing data were minimal (<0.2% per variable and 0.3% in combination for all variables). All available data were used for each regression model. Analyses were conducted in Stata, version 15.1 (StataCorp), and 2-sided *P* < .05 was considered to be statistically significant.

## Results

During the study period, 78 430 interRAI-HC first assessments were undertaken in New Zealand, representing 10% of the population 65 years and older; 41% of all assessments were for people 85 years and older.^18^ We excluded 5144 interviewees younger than 65 years. Therefore, 73 286 New Zealanders (mean age, 81.4 years; age range, 65-109 years; 41 641 [56.9%] female) participated in the study, including 73 095 elderly people (age range, 65-99 years) and 191 centenarians (age range, ≥100 years).

Centenarians had a mean (SD) age of 100.9 (1.2) years compared with 81.4 (7.6) years in the elderly cohort. Centenarians were significantly different from the elderly people across a number of domains, including sex (136 centenarians [71.2%] were women compared with 41 488 elderly people [56.8%], overall χ^2^ test *P* < .001), marital status (170 centenarians [89.0%] were widowed compared with 31 554 elderly people [43.2%], overall χ^2^ test *P* < .001), living arrangements (109 centenarians [57.1%] were living alone and 63 [33.0%] with family compared with 31 943 elderly people [43.7%] living alone and 9976 [13.7%] with family, overall χ^2^ test *P* < .001), and family support (179 centenarians [93.7%] felt supported by their family compared with 65 196 elderly people [89.2%], overall χ^2^ test *P* = .046). Depression status also varied between these groups (70.2% of centerarians vs 59.5% of elderly people were free of depression, overall χ^2^ test *P* = .008), with fewer centenarians depressed (57 [29.8%] for all levels of depression) compared with elderly people (29 601 [40.5%]). No differences were found between these groups in terms of race/ethnicity or season of assessment (Table 1).

**Table 1.** Characteristics of the Study Participants^a^

Characteristic	Total	Participants With Loneliness Data	Noncentenarians	Centenarians	Lonely
Age, y					
65-69	5952 (8.1)	5948	NA	NA	1354 (22.8)
70-74	8898 (12.1)	8895	NA	NA	1789 (20.1)
75-79	13 360 (18.2)	13 355	NA	NA	2539 (19.0)
80-84	17 298 (23.6)	17 284	NA	NA	3359 (19.4)
85-89	16 856 (23.0)	16 850	NA	NA	3353 (19.9)
90-94	8807 (23.0)	8804	NA	NA	1806 (20.5)
95-99	1924 (2.6)	1923	NA	NA	335 (17.4)
100-109	191 (0.3)	191	NA	NA	28 (14.7)
Mean (range)	81.4 (65-109)	81.4 (65-109)	81.4 (65-99)	100.9 (100-109)	NA
Sex					
Male	31 576 (43.1)	31 557	31 502 (43.2)	55 (28.8)	5435(17.2)
Female	41 641 (56.9)	41 624	41 488 (56.8)	136 (71.2)	9112 (21.9)
Missing	69	69	NA	NA	NA
Race/ethnicity					
European	63 203 (86.3)	63 178	63 001 (86.3)	177 (92.7)	12 481 (19.8)
Māori	4454 (6.1)	4453	4448 (6.1)	5 (2.6)	914 (20.5)
Pacific	2607 (3.6)	2604	2602 (3.6)	2 (1.0)	472 (18.1)
Asian	2228 (3.0)	2221	2216 (3.0)	5 (2.6)	496 (22.3)
Other (including MELAA)	741 (1.0)	741	739 (1.0)	2 (1.0)	190 (25.6)
Missing	53	53	NA	NA	NA
Season					
Summer	15 440 (21.0)	15 432	15 385 (21.1)	47 (24.6)	3004 (19.5)
Autumn	18 639 (25.4)	18 629	18 587 (25.4)	42 (22.0)	3821 (20.5)
Winter	19 951 (27.2)	19 939	19 886 (27.2)	53 (27.7)	3904 (19.6)
Spring	19 256 (26.3)	19 250	19 201 (26.3)	49 (25.7)	3834 (19.9)
Missing	0	0	NA	NA	NA
Marital status					
Married, civil union, or de facto committed relationship	31 092 (42.5)	31 073	31 060 (42.6)	13 (6.8)	3933 (12.7)
Never married	3754 (5.1)	3751	3745 (5.1)	6 (3.1)	663 (17.7)
Widowed	31 737 (43.4)	31 724	31 554 (43.2)	170 (89.0)	8293 (26.1)
Separated/divorced	5760 (7.9)	5759	5758 (7.9)	1 (0.5)	1515 (26.3)
Other	843 (1.2)	843	842 (1.2)	1 (0.5)	140 (16.6)
Missing	100	100	NA	NA	NA
Family support					
No	7867 (10.7)	7866	7854 (10.8)	12 (6.3)	2061 (26.2)
Yes	65 409 (89.3)	65 375	65 196 (89.2)	179 (93.7)	12 501 (19.1)
Missing	10	9	NA	NA	NA
Living arrangements					
Alone	32 061 (43.7)	32 052	31 943 (43.7)	109 (57.1)	8926 (27.8)
With partner	28 357 (38.7)	28 342	28 333 (38.8)	9 (4.7)	3138 (11.1)
With family	10 047 (13.7)	10 039	9976 (13.7)	63 (33.0)	2092 (20.8)
With others	2821 (3.8)	2817	2807 (3.8)	10 (5.2)	407 (14.4)
Missing	0	0	NA	NA	NA
Depression					
None, DRS score of 0	43 624 (59.5)	43 589	43 455 (59.5)	134 (70.2)	5902 (13.5)
Mild-moderate, DRS scores of 1-7	28 687 (39.1)	28 687	28 633 (39.2)	54 (28.3)	8218 (28.6)
Moderate-severe, DRS scores of 8-14	972 (1.3)	971	968 (1.3)	3 (1.6)	442 (45.5)
Missing	3	3	NA	NA	NA

Abbreviations: DRS, Depression Rating Scale; MELAA, Middle Eastern, Latin American, African; NA, not applicable.

^a^ Data are presented as number (percentage) of participants unless otherwise indicated. All percentages are column percentages except for percentages for lonely, which indicates the percentage of respondents in that group who were lonely.

Table 1 gives descriptive data for all participants by centenarian status as well as the percentage of lonely people for each level of the variables. Table 2 gives the 3 sets of regression models used in the study: unadjusted, partially adjusted, and fully adjusted.

**Table 2.** Regression Models

Variable	Unadjusted (n ≤73 250)		Partially Adjusted (n = 73 128)		Fully Adjusted (n = 73 079)	
	RR (95% CI)	*P* Value	RR (95% CI)	*P* Value	RR (95% CI)	*P* Value
Differences in risk between those aged 100.9 y and 81.4 y	0.78 (0.67-0.92)	.002	0.75 (0.64-0.88)	<.001	0.68 (0.58-0.79)	<.001
Sex						
Male	0.79 (0.76-0.81)	<.001	0.78 (0.76-0.81)	<.001	1.00 (0.97-1.03)	.84
Female	1 [Reference]		1 [Reference]		1 [Reference]	
Ethnicity						
European	1 [Reference]	<.001	1 [Reference]	<.001	1 [Reference]	<.001
Māori	1.04 (0.98-1.10)		1.00 (0.95-1.07)		1.02 (0.96-1.08)	
Pacific	0.92 (0.84-1.00)		0.90 (0.83-0.98)		1.06 (0.97-1.15)	
Asian	1.13 (1.04-1.22)		1.12 (1.04-1.21)		1.29 (1.20-1.40)	
Other (including MELAA)	1.30 (1.15-1.47)		1.28 (1.13-1.45)		1.31 (1.16-1.47)	
Season						
Summer	1 [Reference]	.06	1 [Reference]	.06	1 [Reference]	.06
Autumn	1.05 (1.01-1.10)		1.05 (1.01-1.10)		1.05 (1.01-1.10)	
Winter	1.01 (0.96-1.05)		1.01 (0.96-1.05)		1.01 (0.97-1.05)	
Spring	1.02 (0.98-1.07)		1.03 (0.98-1.07)		1.03 (0.98-1.15)	
Marital status						
Married, civil union, or de facto committed relationship	1 [Reference]	<.001	NA	NA	1 [Reference]	<.001
Never married	1.40 (1.30-1.51)		NA		0.67 (0.62-0.74)	
Widowed	2.07 (2.00-2.14)		NA		1.06 (1.00-1.12)	
Separated/divorced	2.08 (1.97-2.19)		NA		0.92 (0.86-0.99)	
Other	1.31 (1.12-1.53)		NA		0.63 (0.54-0.74)	
Family support						
No	1 [Reference]	<.001	NA	NA	1 [Reference]	<.001
Yes	0.73 (0.70-0.76)		NA		0.83 (0.79-0.86)	
Living arrangements						
Alone	1 [Reference]	<.001	NA	NA	1 [Reference]	<.001
With partner	0.40 (0.38-0.41)		NA		0.37 (0.35-0.40)	
With family	0.75 (0.72-0.78)		NA		0.71 (0.68-0.74)	
With others	0.52 (0.47-0.57)		NA		0.49 (0.45-0.54)	
Depression						
None, DRS score of 0	1 [Reference]	<.001	NA	NA	1 [Reference]	<.001
Mild-moderate, DRS scores of 1-7	2.12 (2.05-2.18)		NA		2.17 (2.11-2.24)	
Moderate-severe, DRS scores of 8-14	3.36 (3.13-3.62)		NA		3.45 (3.22-3.70)	

Abbreviations: DRS, Depression Rating Scale; MELAA, Middle Eastern, Latin American, African; NA, not applicable; RR, risk ratio.

In an unadjusted model using a third-degree polynomial (*P* < .001 for each of linear, quadratic, and cubic age), the association between loneliness and age showed a 22% (95% CI, 8%-33%; *P* = .002) lower risk of loneliness for those aged 100.9 years compared with those aged 81.4 years (RR, 0.78; 95% CI, 0.67-0.92). In the partially adjusted model, the difference between those aged 100.9 years and those aged 81.4 years was higher, with a 25% (95% CI, 12%-36%; *P* < .001) lower risk for those in the centenarian population.

In the fully adjusted model, the association between loneliness and age was larger (32% [95% CI, 21%-42%] lower risk of loneliness for those aged 100.9 years vs 81.4 years; RR, 0.68; 95% CI, 0.58-0.79; *P* < .001), and the association with sex was no longer significant (estimated risk was the same for men and women; RR for men vs women, 1.00; 95% CI, 0.97-1.03; *P* = .84). Race/ethnicity was overall associated with loneliness, although there was no evidence of a significant difference among European, Māori, and Pacific ethnicities. Persons of Asian or other race/ethnicities were at higher risk of loneliness than were Europeans (Asian: 29% higher [95% CI, 20%-40%], *P* < .001; other: 31% higher [95% CI, 16%-47%], *P* < .001). In this model, never being married was associated with a lower risk of loneliness compared with married, civil union, or de facto relationship (33% lower [95% CI, 26%-38%], *P* < .001). The negative association with being widowed that was observed in the unadjusted model was attenuated (RR, 1.06; 95% CI, 1.00-1.12; *P* = .06), and being separated or divorced was associated with lower risk (8% lower [95% CI, 1%-14%], *P* = .03) of loneliness in this model. Family support (17% lower [95% CI, 14%-21%], *P* < .001) and living with others rather than alone (29%-63% reduction, all *P* < .001) were protective against loneliness, and higher levels of depression were associated with higher risk of loneliness (Wald *P* < .001), with those reporting mild-moderate depression 2.17 (95% CI, 2.11-2.24) times more likely to be lonely and those reporting moderate-severe depression 3.45 (95% CI, 3.22- 3.70) times more likely to be lonely. Time of assessment (season) was not associated with loneliness in any model.

**Figure 1.**
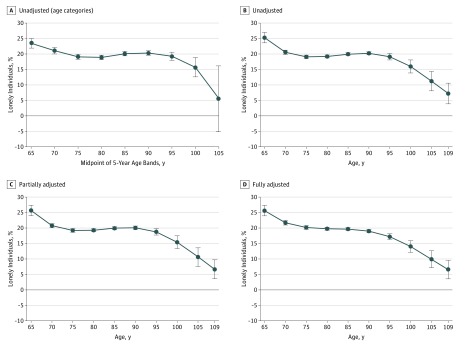
Association Between Loneliness and Age A, Five-year age bands (unadjusted). B, Unadjusted cubic age. C, Cubic age adjusted for potentially modifiable variables. D, Cubic age adjusted for all variables. Error bars indicate 95% CIs.

Figure 1 shows the associations between age and loneliness described above. In the unadjusted models, loneliness decreased from the age of 65 years until 75 years, where it plateaued until approximately 95 years of age, when it started to decrease again. This pattern remained evident in the 2 adjusted models.

From models that examined the modification of associations by sex by adding interactions to the fully adjusted model, the association of loneliness with marital status varied by sex; being widowed or divorced/separated was associated with a greater risk of loneliness in men (widowed: RR, 1.25 [95% CI, 1.16–1.35]; divorced/separated: RR, 1.03 [95% CI, 0.95-1.13]) compared with women (widowed: RR, 0.94 [95% CI, 0.88-1.01]; divorced/separated: RR, 0.83 [95% CI, 0.76–0.90]; for both interactions *P* < .001). For women, there was no evidence of an association of loneliness with being widowed, and there was evidence of a positive association with being divorced or separated (17% lower [95% CI, 10%-24%], *P* < .001). For men, there was no evidence of an association of loneliness with being divorced or separated, but being widowed was associated with greater risk of loneliness (25% higher [95% CI, 16%-35%], *P* < .001). There was no evidence that family support was associated with loneliness differently between men and women. Loneliness among men had a different association with living arrangements (living with partner: RR, 0.33; 95% CI, 0.30-0.35) compared with among women (living with partner: RR, 0.44; 95% CI, 0.41-0.47) (interaction *P* < .001). Although women living with a partner had lower risk compared with women living alone (56% lower [95% CI, 53%-59%], *P* < .001), men living with a partner had even lower risk compared with men living alone (67% lower [95% CI, 65%-70%], *P* < .001).

**Figure 2.**
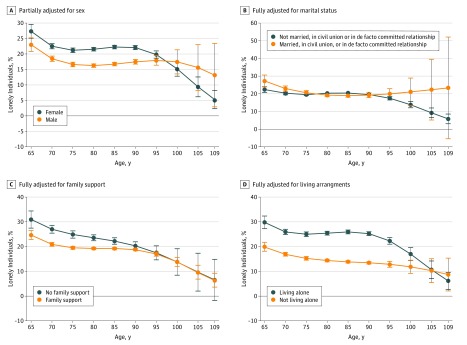
Modification of the Association Between Loneliness and Age Error bars indicate 95% CIs.

Figure 2 shows the modification of associations by sex, marital status, family support, and living arrangements with age. From models that examined the effect modification by age (Figure 2), marital status was collapsed into married, civil union, and de facto committed relationship compared with never married, widowed, or separated/divorced (excluding other because the interpretation of this was unclear), and living arrangements were collapsed into living alone vs all other responses to avoid limited numbers of people within levels of these variables in the centenarian age range.

In the partially adjusted model, at younger ages, women were at greater risk of loneliness than men, a situation that reversed at older ages (Figure 2A), but this difference was no longer evident in the fully adjusted model. The other 3 interactions were all for the fully adjusted model. Figure 2B shows that not being married was protective at younger ages but not afterward. The association of family support diminished among those of older age (Figure 2C). The greater risk associated with living alone also diminished among those of older age (Figure 2D).

## Discussion

Loneliness, defined as “a distressing discrepancy between desired and actual levels of social contact,”^19^^(p1853)^ affects people of all ages. Recent research has recognized loneliness as a public health hazard because of its association with a wide range of conditions, including hypertension, cardiovascular disease, cerebrovascular disease, Alzheimer disease, depression, and insomnia.^1^ The association between loneliness and poor health is unclear with regard to which factor comes first, how loneliness and poor health interact, and whether unknown confounding variables also play a role.^19^

Our findings suggest that in this New Zealand population of older people seeking support, centenarians are less likely to be lonely compared with elderly people. Rates of self-reported loneliness were lower among the centenarians in our study than in previously published research.^5,6,7,8,9^ One explanation is that we sampled only centenarians dwelling in the community. Our findings could also be associated with family support and relative lack of depression among centenarians. Family relationships, especially with children, were found to positively, although indirectly, influence loneliness in a large study of Greek centenarians.^5^ However, different studies have used different instruments, and we cannot rule out these instruments having different sensitivities and specificities for loneliness.

Studies focusing on sex and loneliness in old age report conflicting findings. In our study, the risk of loneliness initially appeared to be lower in males; however, in the fully adjusted model, sex was no longer significant. Family support and living with others was associated with the risk of loneliness, which was 17% lower for family support and 29% to 63% lower for living with others, and there was no evidence that family support was associated with loneliness differently for men and women. Living arrangements, however, had different associations with the risk of loneliness for each sex. Both women and men living with a partner had lower risk of loneliness compared with living alone, but this was more pronounced for men, whose risk was 67% lower compared with 56% for women. In addition, the associations with marital status varied by sex. Men who were widowed or divorced or separated had a greater risk of loneliness, whereas for women, there was no evidence of an association with being widowed.

A large Norwegian study^20^ that compared loneliness in different age groups between 18 and 81 years reported a similar prevalence of loneliness among women and men, as did an Indian study.^21^ A Japanese study^22^ that aimed to explore sex differences in loneliness among persons aged 50 to 70 years reported that loneliness was more prevalent among men than among women. Finally, the Newcastle 85+ Study^23^ reported recently that women spent more time alone than men and reported more loneliness both currently and compared with in the past; a German study reported higher levels of loneliness in women 85 years and older^24^; and a Swedish study reported double the rates of loneliness in elderly women.^25^

Most study participants were European New Zealanders; this group composed 86.3% of our sample, reflecting the ethnic distribution of older New Zealanders,^26^ although the older population is increasing in ethnic diversity.^26,27^ We found that people of Asian and other ethnicities, who composed only 4.0% of the study population, were significantly lonelier than people of other ethnicities. This finding is supported by other research on the association between race/ethnicity and aging and social isolation in New Zealand.^27^ Older Asian immigrants to New Zealand report feeling invisible in their new communities.^28^

Depression is a common condition that has important consequences for older adults; however, few studies have examined depression in centenarians. The prevalence of depression in a sample of Portuguese centenarians was as high as 51% in frail centenarians, but it was 0% in robust centenarians.^29^ This finding is consistent with the high rates of depression-free centenarians in our sample.

### Strengths and Limitations

The strengths of this study include a near-complete data set for this well-defined population, with low rates of missing data, and reasonably precise CIs from the large sample size. There are several limitations to the present study. Loneliness was assessed using a single-item question, although the interRAI-HC is an internationally validated screening tool. Cohort effects were inevitable because of the cross-sectional nature of the study; the age range was more than 40 years in our sample. Cohort effects may have significant consequences on the differences between the youngest and oldest people in our study because these participants will have experienced different socioeconomic and political environments during their lives. However, in terms of screening for loneliness for the purpose of targeting interventions for those most likely to be lonely, this cohort effect is irrelevant. The interRAI-HC assessments are undertaken in New Zealand for people 65 years or older with an age-related disability that is affecting their function, is likely to continue for a minimum of 6 months, and creates a potential need for support services. Our community-based sample of people whose frailty necessitated undertaking an interRAI-HC assessment is likely to not represent all elderly people, and it would be worthwhile to replicate these findings in the general population. However, because the interRAI-HC is more likely to capture people older than 85 years, the comparisons of centenarians with younger people may be more representative of findings in the older general population.

## Conclusions

Our sample of New Zealand centenarians who were assessed for support were less lonely than other groups studied internationally. Although other researchers reported an increase in loneliness as people age,^23,30^ we found evidence of a significant negative association between loneliness and age in unadjusted models and after adjustment for a range of demographic and experiential variables. We found evidence that depression, family support, and living arrangements were psychosocial variables that were associated with centenarians’ risk of loneliness. Knowing these variables may help us address risk factors for loneliness in elderly people. One of the important questions still unresolved is the trajectory of loneliness along the life cycle and differences in prevalence of loneliness in different settings.^23,30^
